# Psychosocial effects of COVID-19 pandemic in Bolivia. Preliminary results

**DOI:** 10.1192/j.eurpsy.2021.841

**Published:** 2021-08-13

**Authors:** D. Valdés, F. Molina, C. Barrientos, P. Valenzuela, A. Basaigoitia, M. Burrone, G. Reginatto, I. Leniz, M. Solis-Soto

**Affiliations:** 1 Instituto De Ciencias De La Salud, Universidad de O’Higgins, Rancagua, Chile; 2 Consulting Office, Salud Global, Sucre, Bolivia; 3 Dirección De Asuntos Estudiantiles, Universidad de O´Higgins, Rancagua, Chile

**Keywords:** COVID-19, Bolivia, psychosocial, mental health

## Abstract

**Introduction:**

The global health crisis due to Coronavirus Disease 2019 (COVID-19) and related containment measures have led to changes in daily life and, therefore, social and psychological impacts on the population.

**Objectives:**

To explore the psychological and social impact of COVID-19 in the general population of Bolivia.

**Methods:**

Cross-sectional study was implemented using an anonymous and self-administered online questionnaire. Adult people were invited to participate through social networks between May to June 2020. The questionnaire included sociodemographic information, coping strategies, changes in income and working conditions and psychological distress (K10 Scale).

**Results:**

A total of 878 adults living in Bolivia answered the questionnaire. Most people considered COVID-19 as a quite/very serious health problem that affects the entire population, without distinction. 65% reported to accomplish lock down measure, however, one of the main reasons for non-compliance is the need to go out to work. Half of participants (50%) reduced worked hours and 18% modified their employment contract. However, 70% reduced household income. A considerable percentage (62%) reported psychological distress (46% with moderate or severe). It was higher in women, young people and among those with lower household income. In addition, social networks and watching series and movies were the main coping strategies reported.

**Conclusions:**

The COVID-19 pandemic has a considerable impact on psychological and social level. The negative impact was greater in some population groups such as women, young people, and those with a lower socioeconomic level, which may further increase inequities.

